# Supplementary material for: Albumin change predicts failure in ulcerative colitis treated with adalimumab
Source: PLoS One. 2024 Jan 2;19(1):e0295681. doi: 10.1371/journal.pone.0295681 (PMC10760906; doi:10.1371/journal.pone.0295681)
Supplement: S1 Table — (DOCX) [file pone.0295681.s001.docx]

| Variable | Failure | Non-failure | P-value |
| --- | --- | --- | --- |
|  | n=13 | n = 19 |  |
| Neutrophile count (/μL), median [IQR] | 6,513 [3,771–7,000] | 5,115 [3,437–8,037] | 0.893 |
| Neutrophile rate (%), median [IQR] | 72.0 [65.9–76.0] | 66.1 [59.4–79.0] | 0.631 |
| Lymphocyte count (/μL), median [IQR] | 1,324 [802–1,933] | 1,348 [1,028–1,919] | 0.715 |
| Lymphocyte rate (%), median [IQR] | 17.0 [12.0–20.7] | 20.2 [13.7–31.2] | 0.309 |
| Monocyte count (/μL), median [IQR] | 527 [278–1037] | 379 [205–567] | 0.478 |
| Monocyte rate (%), median [IQR] | 6.8 [4.6–8.3] | 5.2 [4.3–6.2] | 0.205 |
| NLR, median [IQR] | 4.4 [3.4–6.0] | 3.6 [1.9–5.5] | 0.328 |
| NMR, median [IQR] | 10.8 [7.5–14.3] | 12.7 [9.7–15.4] | 0.478 |
| LMR, median [IQR] | 3.1 [2.0–5.1] | 4.8 [3.0–5.6] | 0.25 |

IQR, interquartile range; NLR neutrophil to lymphocyte ratio; NMR, neutrophil to monocyte ratio; LMR, lymphocyte to monocyte ratio.
