# Supplementary material for: Albumin change predicts failure in ulcerative colitis treated with adalimumab
Source: PLoS One. 2024 Jan 2;19(1):e0295681. doi: 10.1371/journal.pone.0295681 (PMC10760906; doi:10.1371/journal.pone.0295681)
Supplement: S2 Table — (DOCX) [file pone.0295681.s002.docx]

| Variable | Failure | Non-failure | P-value |
| --- | --- | --- | --- |
|  | N = 10 | N = 21 |  |
| Alb at week 6 (g/dL), median [IQR] | 4.3 [3.8–4.4] | 4.3 [4.0–4.6] | 0.525 |
| CRP at week 6, median [IQR] | 0.04 [0.02–0.25] | 0.05 [0.03–0.19] | 0.700 |
| WBC at week 0 (/µL), median [IQR] | 5,705 [4,947–6,647] | 5,840 [5,030–7,740] | 0.833 |
| Hb at week 6 (g/dL), median [IQR] | 12.8 [11.6–14.5] | 12.8 [11.7–14.6] | 0.983 |
| Plt at week 6 (×104/µL), median [IQR] | 31.5 [25.9–33.5] | 25.6 [20.2–32.2] | 0.410 |

Alb, albumin; IQR, interquartile range; CRP, C-reactive protein; WBC, white blood cell; Hb, hemoglobin; Plt, platelet
