# Supplementary material for: Albumin change predicts failure in ulcerative colitis treated with adalimumab
Source: PLoS One. 2024 Jan 2;19(1):e0295681. doi: 10.1371/journal.pone.0295681 (PMC10760906; doi:10.1371/journal.pone.0295681)
Supplement: S3 Table — (DOCX) [file pone.0295681.s003.docx]

|  | | Overall N = 25 | Failure N = 10 | Non-failure N = 15 | P-value |
| --- | --- | --- | --- | --- | --- |
| Age (year), median [IQR] | | 46 [31–56] | 35 [23–56] | 47 [41–55] | 0.244 |
| Male/Female, n (%) |  | 12 (48.0) /13 (52.0) | 3 (30.0) /7 (70.0) | 9 (60.0) /6 (40.0) | 0.226 |
| Disease duration (year), median [IQR] | | 9 [3–15] | 5 [2–9] | 13 [5–16] | 0.085 |
| Disease extent, n (%) | Extensive colitis | 21 (84.0) | 8 (80.0) | 13 (86.7) | 0.730 |
|  | Left-sided colitis | 3 (12.0) | 2 (20.0) | 1 (6.7) |  |
|  | Proctitis | 1 (4.0) | 0 (0.0) | 1 (6.7) |  |
| CAI (Rachmilewitz index), median [IQR] | | 9 [7–11] | 8 [7–10] | 9 [7–11] | 0.955 |
| MES, n (%) | MES 1 | 2 (8.0) | 1 (10.0) | 1 (6.7) | 0.487 |
|  | MES 2 | 14 (56.0) | 4 (40.0) | 10 (66.7) |  |
|  | MES 3 | 9 (36.0) | 5 (50.0) | 4 (26.7) |  |
| UCEIS, median [IQR] |  | 5 [1–8] | 5 [2–8] | 5 [1–7] | 0.778 |
| Other medication, n (%) | Oral 5-ASA | 15 (60.0) | 5 (50.0) | 10 (66.7) | 0.442 |
|  | Suppository steroids | 2 (8.0) | 0 (0.0) | 2 (13.3) | 0.500 |
|  | Systemic steroids | 8 (32.0) | 3 (30.0) | 5 (33.3) | 1 |
|  | Immunomodulator | 7 (28.0) | 3 (30.0) | 4 (26.7) | 1 |
|  | Tacrolimus | 1 (4.0) | 0 (0.0) | 1 (6.7) | 1 |
| History of biologicals use |  | 5 (20.0) | 2 (20.0) | 3 (20.0) | 1 |

IQR, interquartile range; CAI, clinical activity index; MES, Mayo endoscopic subscore; UCEIS, ulcerative colitis endoscopic index of severity; 5-ASA, 5-aminosalicylic acid
