# Supplementary material for: Albumin change predicts failure in ulcerative colitis treated with adalimumab
Source: PLoS One. 2024 Jan 2;19(1):e0295681. doi: 10.1371/journal.pone.0295681 (PMC10760906; doi:10.1371/journal.pone.0295681)
Supplement: S4 Table — (DOCX) [file pone.0295681.s004.docx]

|  | | Overall | Failure | Non-failure | P-value |
| --- | --- | --- | --- | --- | --- |
|  |  | N = 68 | N = 20 | N = 48 |  |
| ADA/GLM/IFX | | 34 (50.0) / 20 (29.4) / 14 (20.6) | 13 (65.0) / 3 (15.0) / 4 (20.0) | 21 (43.8) / 17 (35.4) / 10 (20.8) | 0.204 |
| Age (year), median [IQR] | | 46 [32, 58] | 43 [23, 62] | 47 [33, 56] | 0.829 |
| Male / Female, n (%) | | 39 (57.4) / 29 (42.6) | 12 (60.0) / 8 (40.0) | 27 (56.2) / 21 (43.8) | 1 |
| Disease duration (year), median [IQR] | | 5 [2, 11] | 3 [1, 7] | 7 [2, 13] | 0.220 |
| Disease extent, n (%) | Extensive colitis | 55 (80.9) | 15 (75.0) | 40 (83.3) | 0.340 |
|  | Left sided colitis | 11 (16.2) | 5 (25.0) | 6 (12.5) |  |
|  | Proctitis | 2 (2.9) | 0 (0.0) | 2 (4.2) |  |
| CAI (Rachmilewitz index), median [IQR] | | 6 [4, 9] | 7 [4, 10] | 6 [4, 9] | 0.201 |
| MES, n (%) | MES 0 | 2 (2.9) | 1 (5.0) | 1 (2.1) | 0.369 |
|  | MES 1 | 7 (10.3) | 2 (10.0) | 5 (10.4) |  |
|  | MES 2 | 30 (44.1) | 6 (30.0) | 24 (50.0) |  |
|  | MES 3 | 29 (42.6) | 11 (55.0) | 18 (37.5) |  |
| UCEIS, median [IQR] | | 5 [3, 6] | 5 [3, 6] | 5 [3, 6] | 0.469 |
| Other medication, n (%) | Oral 5-ASA | 32 (47.1) | 8 (40.0) | 24 (50.0) | 0.595 |
|  | Suppository steroids | 2 (2.9) | 0 (0.0) | 2 (4.2) | 1 |
|  | Systemic steroids | 22 (32.4) | 8 (40.0) | 14 (29.2) | 0.407 |
|  | Immunomodulator | 22 (32.4) | 5 (25.0) | 17 (35.4) | 0.571 |
|  | Tacrolimus | 11 (16.2) | 5 (25.0) | 6 (12.5) | 0.279 |
| History of biologicals use | | 24 (35.3) | 7 (35.0) | 17 (35.4) | 1 |

ADA, adalimumab; GLM, golimumab; IFX, infliximab; IQR, interquartile range; CAI, clinical activity index; MES, Mayo endoscopic subscore; UCEIS, ulcerative colitis endoscopic index of severity; 5-ASA, 5-aminosalicylic acid
