# Supplementary material for: Albumin change predicts failure in ulcerative colitis treated with adalimumab
Source: PLoS One. 2024 Jan 2;19(1):e0295681. doi: 10.1371/journal.pone.0295681 (PMC10760906; doi:10.1371/journal.pone.0295681)
Supplement: S1 File — (PDF) [file pone.0295681.s006.pdf]

様式6

2021年04月07日

審査結果通知書

申請者(研究責任者)
杉本 健　殿

浜松医科大学長

医学系研究に関する審査結果を、下記のとおり、通知します。

記

研究番号	21-029
研究課題名	炎症性腸疾患に対する生物学的製剤の血液検査学的な有効性の検討
審査事項	■医学系研究の実施の可否<br>□医学系研究の継続の可否<br>□医学系研究に関する変更<br>□重篤な有害事象<br>□継続審査<br>□その他<br>□その他
審査区分	□本審査<br>■迅速審査(審査終了日:西暦2021年03月31日)
審査結果	■承認<br>□条件付き承認<br>□再提出<br>□不承認<br>□非該当<br>□既承認事項の取り消し
備考	
