# Supplementary material for: Albumin change predicts failure in ulcerative colitis treated with adalimumab
Source: PLoS One. 2024 Jan 2;19(1):e0295681. doi: 10.1371/journal.pone.0295681 (PMC10760906; doi:10.1371/journal.pone.0295681)
Supplement: S2 File — (PDF) [file pone.0295681.s007.pdf]

Form 6

April 7, 2021

Review result notification

Applicant (Research director)
Dr. Ken Sugimoto

President of Hamamatsu University School of Medicine

The review results regarding medical research is notified as follows.

Summary

Research number	21-029
Research topic name	Examination of blood test efficacy of biological agents for inflammatory bowel disease
Review matters	■Is it possible to conduct medical research?<br>☐Whether or not medical research can be continued<br>☐Changes regarding medical research<br>☐Serious adverse events<br>☐Continuous examination<br>☐Others<br>☐Others
Review category	☐Main review<br>■Expedited review (review end date: March 31, 2021)
Review results	■Approval<br>☐Conditional approval<br>☐Resubmit<br>☐Disapproval<br>☐Not applicable<br>☐Cancellation of approved items
Remarks	
